# The Attitude of Great Writers towards Disease

**Published:** 1892-07-16

**Authors:** 


### THE ATTITUDE OF GREAT WRITERS TOWARDS DISEASE.

### V.—LORD TENNYSON.

MANY are the forms under which the suffering of human life unfolds itself to the poet. For pain is the poet's dower, and he must feel the great throb of the world's anguish in the compass of his own being before he can check its beats and interpret its symptoms to his fellow men. Suffering is the great master of them all, but infinite as nature herself are the methods by which suffering schools her disciples.

Very early in life our great living poet marked for his own the crown of sorrow which waits round the gates of death and passes from the head of the dying to rest with lingering pressure on those who love and live, yet in this poet who has won his laurels in the valley of the shadow of death is no distant tampering with morbid pleasure in disease and decay.

Whatever crazy sorrow saith,
No life that breathes with human breath
Has ever truly longed for death,
'Tis life, whereof our nerves are scant,
Oh life, not death, for which we pant;
More life, and fuller, that I want.

So he sang in early life, and his later work brings no contradiction. It is indeed by virtue of the entire healthiness of his genius that he has been enabled to paint the power and pathos of death in life.

There is a small picture of the Crucifixion by Tintoretto, in Venice, which illustrates something of the method of "In Memoriam." The central Figure is merely outlined in the background and alone, a wide landscape of wonderful beauty stretches to the far distance, grass and flowers and living creatures in the foreground, painted with curious elaboration, and a glowing sunshine over all. Gradually as the scene grows into the mind it becomes clear that the painter has aimed at realising the very pain of death in its human aspect—death in the prime of life, in full strength, untainted by decay, while the meanest forms of nature overflow with that vital essence which in the sufferer is passing consciously drop by drop away.

And so in the requiem the foreground teems with ever-varying life and beauty. One living picture succeeds another, the "lonely seas" give place to the "babbling Wye," bird, beast, and flower, the changing seasons, the familiar scenes of every-day life, all are painted with the sure touch which lends its peculiar grace to whatever it approaches. Yet the dim figure in the background can never be overlooked, and the detail is only thus lovingly elaborated that it may serve as foil to the unseen presence of death.

It is this love of life and of every living creature, which lends the poet power to wrestle with death. Nothing at all akin to that great conflict exists in any language. No one ever went for consolation to Milton's lament, "faultily faultless, icily regular," over his friend. Assuredly none ever found a stay of comfort in Shelley's stirring words of vindication over Keats. But "In Memoriam" is in the hearts of us all, lying open on the knee in sacred seasons of grief, read one to another on peaceful summer afternoons, far from "the dust, and din, and steam of town," when memory wakes "not all regret"; always a quiet friend with soothing presence, lifting by gentle pressure of sympathy to higher consolation.

The pain of separation which finds its full expression in "In Memoriam," mingles in many other poems.

For this alone on Death I wreak
The wrath that garners in my heart;
He put our lives so far apart
We cannot hear each other speak.

In "Enoch Arden," in "Maud," in "Elaine," in "Queen Mary," the ache of parting foreshadows and passes into the ache of death.

Due perhaps to the deep and spiritual aspect under which death has been most vividly present to the Laureate is his very marked avoidance of the horrors commonly attendant upon violent death. Had he written fewer battle scenes this might not have been perceptible. But a very strong distaste for carnage is revealed by a comparison of the battles, tournaments, and duels which are necessarily many, in the "Idylls of the King," with the prose of Sir Thomas Mallory from which they are adapted. A good deal of blood is shed, but as a kind of painful necessity; the killing is done on the principle of inflicting as little pain as possible, and the injuries received strike no thrill of horror as in the Homeric battle. If this be a flaw, it is one which in some eyes approaches perilously near an added beauty. But if the fierce realism of the old writers is gratefully missed, it must be admitted that with the unpleasant detail much of the spirit of battle has departed also. The lust of war is wanting—the passion for killing which urged on the old heroes, and its place is supplied by complex motives which smell of the study rather than the battle-field.

Once only the ghastly realities of war are painted in the stirring poem on the Relief of Lucknow:—

Ever the day with its traitorous death from the loopholes around,
Ever the night with its coffinless corpse to be laid in the ground,
Heat like the mouth of a hell, or a deluge of cataract skies,
Stench of old offal decaying, and infinite torment of flies,
Thoughts of the breeze of May blowing over an English field,
Cholera, scurvy, and fever, the wound that *would* not be healed,
Lopping away of the limb by the pitiful—pitiless knife,
Torture and trouble in vain, for it never could save us a life.
Valour of delicate women who tended the hospital bed,
Horror of women in travail among the dying and dead;
Grief for our perishing children, and never a moment for grief,
Toil and ineffable weariness . . . .

After this we cannot forbear quoting a hospital scene of a very different nature. He who wrote long years ago

Ring out old shapes of foul disease, has been a never-failing witness against the tyranny both of oppression and neglect, and no modern scheme of mercy "dear to the man that is dear to God" has lacked his sympathy. A very tender pathos lies in the nurse's tale of her little patient, who overhears, while half asleep, the doctor's verdict:—

"Nurse, I must do it to-morrow; she'll never live through it, I fear."
Never since I was nurse had I been so grieved and so vexed!
Emmie had heard him. Softly she called from her cot to the next,
"He says I shall never live through it, O Annie, what shall I do?"
Annie considered. "If I," said the wise little Annie, "was you,
I should cry to the dear Lord Jesus to help me, for, Emmie, you see,
It's all in the picture there: little children should come to Me."
"Yes, and I will," said Emmie, "but then if I call to the Lord,
How should He know that it's me? Such a lot of beds in the ward!"
That was a puzzle for Annie. Again she considered and said,
"Emmie, you put out your arms, and you leave 'em outside on the bed,
The Lord has so much to see to; but Emmie, you tell it Him plain,
It's the little girl with her arms lying out on the counterpane."
The nurse is forced to leave her for the night. Morning dawns,
And the doctor came at his hour, and we went to see to the child.

He had brought his ghastly tools; we believed her asleep again—
Her dear, long, lean, little arms lying out on the counterpane
Say that His day is done! Ah, why should we care what they say?
The Lord of the children had heard her, and Emmie had passed away.

Here, then, is death in its highest and holiest aspect. And the poet's last utterance, clothed in a lingering sweetness which places it in the memory high among all treasures gathered from the same loved store, shows us death still as the same gracious presence.

And though beyond this bourne of time and space
The tide may bear me far,
I hope to see my Pilot face to face
When I have crossed the bar.

